# PRO-FIBROTIC PHENOTYPE OF BONE MARROW STROMAL CELLS IN MODIC TYPE 1 CHANGES

**DOI:** 10.22203/eCM.v041a42

**Published:** 2021-06-08

**Authors:** I. Heggli, S. Epprecht, A. Juengel, R. Schuepbach, N. Farshad-Amacker, C. German, T. Mengis, N. Herger, L. Straumann, S. Baumgartner, M. Betz, J.M. Spirig, F. Wanivenhaus, N. Ulrich, D. Bellut, F. Brunner, M. Farshad, O. Distler, S. Dudli

**Affiliations:** 1 Center of Experimental Rheumatology, Department of Rheumatology, University Hospital, University of Zurich, Zurich, Switzerland; 2 Department of Physical Medicine and Rheumatology, Balgrist University Hospital, University of Zurich, Zurich, Switzerland; 3 Unit of Clinical and Applied Research, Balgrist University Hospital, University of Zurich, Zurich, Switzerland; 4 Department of Radiology, Balgrist University Hospital, University of Zurich, Zurich, Switzerland; 5 Department of Orthopaedic Surgery, Balgrist University Hospital, University of Zurich, Zurich, Switzerland; 6 Department of Neurosurgery, University Hospital Zurich, Zurich, Switzerland

**Keywords:** Modic change, fibrosis, bone marrow stromal cells, mesenchymal stem cell, extracellular matrix, cell differentiation, cell contractility, cell adhesion, focal adhesion kinase, pro-fibrotic phenotype

## Abstract

Modic type 1 changes (MC1) are painful vertebral bone marrow lesions frequently found in patients suffering from chronic low-back pain. Marrow fibrosis is a hallmark of MC1. Bone marrow stromal cells (BMSCs) are key players in other fibrotic bone marrow pathologies, yet their role in MC1 is unknown. The present study aimed to characterise MC1 BMSCs and hypothesised a pro-fibrotic role of BMSCs in MC1.

BMSCs were isolated from patients undergoing lumbar spinal fusion from MC1 and adjacent control vertebrae. Frequency of colony-forming unit fibroblast (CFU-F), expression of stem cell surface markers, differentiation capacity, transcriptome, matrix adhesion, cell contractility as well as expression of pro-collagen type I alpha 1, α-smooth muscle actin, integrins and focal adhesion kinase (FAK) were compared.

More CFU-F and increased expression of C-X-C-motif-chemokine 12 were found in MC1 BMSCs, possibly indicating overrepresentation of a perisinusoidal BMSC population.

RNA sequencing analysis showed enrichment in extracellular matrix proteins and fibrosis-related signalling genes. Increases in pro-collagen type I alpha 1 expression, cell adhesion, cell contractility and phosphorylation of FAK provided further evidence for their pro-fibrotic phenotype. Moreover, a leptin receptor high expressing (LEPR^high^) BMSC population was identified that differentiated under transforming growth factor beta 1 stimulation into myofibroblasts in MC1 but not in control BMSCs.

In conclusion, pro-fibrotic changes in MC1 BMSCs and a LEPR^high^ MC1 BMSC subpopulation susceptible to myofibroblast differentiation were found. Fibrosis is a hallmark of MC1 and a potential therapeutic target. A causal link between the pro-fibrotic phenotype and clinical characteristics needs to be demonstrated.

## Introduction

MC are vertebral bone marrow lesions visualised by MRI that occur around a degenerated intervertebral disc (Modic et al., 1988). Of the three interconvertible types of MC, MC1 has the highest association with LBP (Kääpä et al., 2012; Nguyen et al., 2015). LBP patients with MC1 report a higher frequency and duration of LBP episodes, seek care more often and have a higher risk for no improvement in pain and function (Chung et al., 2004; Jensen et al., 2008; Jensen et al., 2014; Schistad et al., 2014; Sørile et al., 2012). Larger lesions are more painful and have a positive predictive value for discography concordant pain of up to 100 % (Järvinen et al., 2015; Weishaupt et al., 2001). In contrast to disc herniation, where nerve compression causes back and leg pain, MC pain originates from the endplate and vertebra (Fields et al., 2014; Lotz et al., 2013; Ohtori et al., 2006).

Despite the high clinical and socioeconomic importance, MC1 pathophysiology is largely unknown. Only a few studies have investigated human surgical MC1 bone marrow tissue. In Michael Modic’s original paper, he described MC1 bone marrow based on three biopsies as a fibrovascular granulation tissue (Modic et al., 1988). Perilli et al. (2015) investigated nine MC1 biopsies by histology and micro-CT and concluded that MC1 had a high bone turnover possibly due to inflammation. In a cross-sectional analysis of MC1 bone marrow aspirates and of adjacent discs, Dudli et al. (2017) found pro-fibrotic changes (*i.e.* increased *COL1A1* expression) in MC1 and a pro-fibrotic and pro-inflammatory cross-talk between MC1 bone marrow and the adjacent disc. In summary, these studies have identified fibrosis, inflammation and high bone turnover as the pathophysiological hallmarks of MC1. However, the fibrotic mechanism in MC1 remains largely unknown.

BMSCs are key regulators of bone marrow inflammation, fibrosis and bone turnover (Kondo et al., 2001; Schneider et al., 2017; Ziegler et al., 2016). BMSCs fulfil the criteria for MSCs (Dominici et al., 2006): they express CD73, CD90, CD105 but not CD14, CD19, CD34, CD45 and HLA-DR; they form CFU-F on plastic dishes and can differentiate into adipocytes, osteoblasts and chondrocytes. Primary BMSCs are a heterogeneous population and their reported markers include LEPR, CD54, CD106, CD140a, CD146, CD271, CXCL12, nestin and NG2 (El Agha et al., 2017a; Decker et al., 2017). Expression of specific markers has been associated with their bone marrow niche localisation. BMSCs from the endosteal niche are CD146^−^ while BMSCs from the vascular niche express CD106, CD146, CD271, CXCL12 and NG2 (Barilani et al., 2018; Tormin et al., 2011). Within the vascular niche, periarteriolar and perisinusoidal BMSCs can be distinguished based on their expression profile. Nestin expression is higher in periarteriolar BMSCs, while LEPR and CD106 is higher in sinusoidal BMSCs (El Agha et al., 2017a; Jacobsen et al., 1996). Expression of specific markers has also been linked to pathological conditions. For example, perivascular, LEPR-expressing BMSCs have been identified as the cellular origin of collagen-producing myofibroblasts in bone marrow fibrosis (Decker et al., 2017). These cells are profoundly dysregulated as evidenced by myelofibrosis, osteosclerosis and neoangiogenesis. Perivascular BMSCs are pericyte-like cells and Notch signalling promotes their PMT. Notch signalling is an important pro-fibrotic pathway affecting BMSC proliferation and differentiation (Azizidoost et al., 2015; Dong et al., 2010; Wang et al., 2019).

Despite the important role of BMSCs in fibrosis, their role in MC1 is unknown. To gauge the role of BMSCs in MC1, MC1 BMSCs were compared to intra-patient control BMSCs from chronic LBP patients undergoing spinal fusion. The aim of the study was to identify and characterise potential pro-fibrotic attributes of MC1 BMSCs.

## Material and Methods

The study was conducted in accordance with the Declaration of Helsinki and approved by the local Ethics Commission (#2017-00761; approved June 05, 2017). Chemicals were purchased from Sigma-Aldrich if not stated otherwise.

### Study subjects

**Fig. 1.**
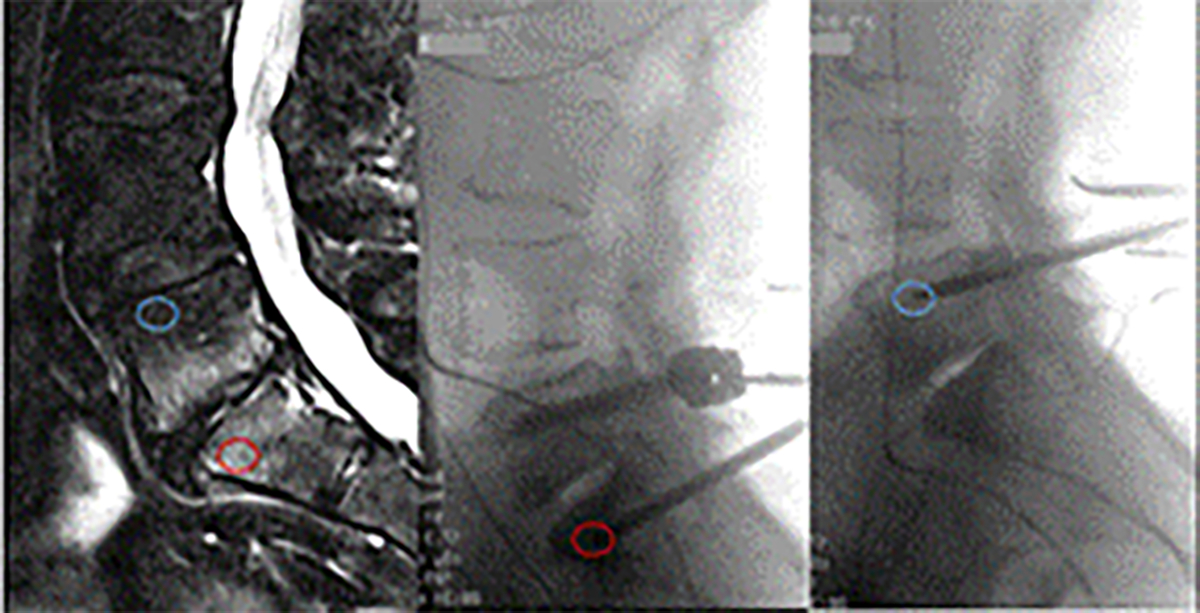
MC1 at L5/S1. Pre-operative sagittal T2w MRI (left), intraoperative sagittal X-ray during harvesting of MC1 aspirate (middle) and intra-patient control aspirate (right). Red and blue circle indicate location of MC1 and control aspirate, respectively.

Informed consent was obtained from each subject. The study included 14 patients undergoing lumbar spondylodesis at the Balgrist University Hospital between April 2018 and July 2020. Inclusion criteria were (i) spinal fusion at level of MC1 with a large enough MC1 lesion that a pedicle screw came to lie into the MC1 lesion, (ii) a second pedicle screw in a vertebral bone marrow region without MC from the same patient (Fig. 1). Exclusion criteria were applied based on past medical history and were infectious diseases, malignancies, prior instrumented back surgery, rheumatic markers *e.g.* HLA, auto-antibodies, juvenile scoliosis and leg pain greater than back pain on VAS. Patients were identified pre-operatively based on T1w, T2w and STIR lumbar MRI. Subjects completed a 10-item ODI questionnaire and indicated intensity of back and leg pain on a 10 cm VAS (VASback and VASleg, respectively). Patient demographics are summarised in Table 1.

Magnetic resonance images were retrospectively analysed by a radiologist with > 12 years of experience including > 5 years in musculoskeletal radiology. The MRI sequences available were sagittal T1w and sagittal T2w images, sagittal STIR, coronal T2w images, intra-operative X-ray as well as post-operative T1w and T2w sagittal images. Mean difference from date of surgery to MRI acquisition was 41.9 ± 29.8 d. The location of the Jamshidi needle tip during aspiration (or pedicle screw tip if no intraoperative X-ray were available) was determined and MC type (no MC, MC1, MC2, MC3) and extent ( < 50 %, > 50 % of vertebral body height) at needle tip location was rated. Total endplate score (0–6) (Rajasekaran et al., 2008) and Pfirmann grade of adjacent disc (0–5) were compared using paired Wilcoxon tests.

### Bone marrow aspirates and BMSC culture

Spondylodesis requires the insertion of pedicle screws into the vertebral bodies. Using the pedicle screw trajectories, bone marrow aspirates were taken before screw insertion using a Jamshidi needle. Two aspirates were taken from each patient: a MC1 and an intra-patient control aspirate (Fig. 1). Proper positioning of the Jamshidi needle was key. In pre-operative discussions with the surgeons and with intra-procedural radiographic guidance, proper needle position was ensured. Surgeons were advised to aspirate 2.5–3.5 mL bone marrow immediately after reaching the target depth. Aspirates were taken with a 10 mL syringe and transferred within 2 min into K2-EDTA tubes (BD Bioscience). Bone marrow aspirates were centrifuged at 700 ×*g* and 4 °C for 15 min. Bone marrow fat and plasma were removed and red blood cells were lysed.

BMSCs were isolated by plastic adherence (Dominici et al., 2006; Hoch and Leach, 2015). 30 × 10^6^ cells were resuspended in growth medium [αMEM without nucleosides (Gibco^™^), 50 U/mL P/S (Gibco), 10 mmol/L HEPES (Gibco), 10 % heat-inactivated FCS and 2.5 ng/mL human bFGF (PeproTech)] and seeded in T75 flasks. Cells were incubated at 37 °C and 5 % CO_2_ with medium change every 3–4 d. For all assays, BMSCs from passage 3–5 were used (Table 2). MC1 BMSCs and control BMSCs were used at the same passage for each patient. The performed assays for each patient are indicated in Table 1.

### Assay 1 and 2: flow cytometric characterisation

In assay 1, stem cell surface markers CD105, CD73, CD90, CD45, CD34, CD14 and CD19 were stained for 45 min at room temperature (Table 3), washed with FACS buffer (PBS without Ca^2+^ and Mg^2+^, 1 % FCS) and analysed using a BD LSRFortessa^™^ Flow Cytometer. Single measures of MC1 and control BMSCs were performed. Ratio of CD73^+^, CD90^+^, CD105^+^, CD45^−^, CD34^−^, CD14^−^, CD19^−^ cells was calculated using FlowJo (version 10.7.1) and compared between MC1 and control BMSCs by paired *t*-test.

In assay 2, for intracellular epitopes (nestin, CXCL12) measurement, cells were permeabilised using BD FACS^™^ Permeabilizing Solution 2 according to manufacturer’s protocol. Cells were stained either for CD54, CD106, CD140a, CD146, CD271, LEPR, NG2, CXCL12 or nestin for 45 min at room temperature (Table 3), washed with FACS buffer and analysed using BD LSRFortessa^™^ Flow Cytometer. Single measures of MC1 and control BMSCs were performed. The non-normal distributed difference of MC1 and control MFI was tested against null hypothesis (*μ*0 = 0) by Wilcoxon test. Pearson correlation coefficients were calculated between different markers.

### Assay 3: differentiation capacity

Adipogenesis was induced in sextuplicate in 80–90 % confluent BMSC layers in 24-well plates with inductive medium (αMEM no nucleosides medium, 50 U/mL P/S, 10 mmol/L HEPES, 10 % FCS, 5 μg/mL insulin, 10^−7^ mol/L dexamethasone, 0.5 mmol/L isobutylmethylxanthine, 60 μmol/L indomethacin). Inductive medium was changed every 3 d. BMSCs were fixed when MC1 and control cells showed fat droplets (after 14–21 d, same day for MC1 and control). Lipid droplets were stained with oil red O solution. After rinsing with 50 % ethanol and distilled water, oil red O was dissolved in 100 % 2-propanol and absorbance was read at 500 nm. Median absorbances of sextuplicate were calculated. MC1 was normalised to control (100 %) and tested against null hypothesis (*μ*0 = 100 %) using one sample *t*-test.

Osteogenesis was induced in sextuplicate of 100 % confluent BMSC layers in 24-well plates with inductive medium (αMEM no nucleosides medium, 50 U/mL P/S, 10 mmol/L HEPES, 10 % FCS, 10^−7^ mol/L dexamethasone, 10 mmol/L β glycerophosphate, 50 μmol/L L-ascorbic acid 2-phosphate). Inductive medium was changed every 3 d. BMSCs were fixed after 21 to 28 d (same day for MC1 and control) and stained with a 2 % alizarin red solution. The staining was dissolved with 10 % cetylpyridiniumchloride solution and absorbance was read at 570 nm. Median absorbances were calculated. MC1 was normalised to control (100 %) and tested against null hypothesis (*μ*0 = 100 %) using one sample *t*-test.

Chondrogenesis was induced in BMSC pellets (0.3 × 10^6^ cells) seeded in sextuplicate on 96-well ultra-low attachment U-bottom plates with inductive medium (DMEM high glucose, 50 U/mL P/S, 10 mmol/L HEPES, 10^−7^ mol/L dexamethasone, 1 % insulin-transferrin-sodium selenite medium supplement, 1 % non-essential amino acid, 0.05 mmol/L L-ascorbic acid-2-phosphate, 10 ng/mL recombinant human TGF-β1). Medium was changed every 3 d. Cells were fixed after 28 d. 2 to 3 pellets were pooled and digested overnight with papain (60 °C, 125 ug/mL dissolved in 5 mmol/L L-cysteine HCl, 5 mmol/L Na-citrate, 150 mmol/L NaCl, 5 mmol/L EDTA, pH 6.0). DNA was quantified using DNA quantification Kit according to manufacturer’s protocol. GAG was quantified using 1,9-dimethylmethylene blue assay (Enobakhare et al., 1996). Median GAG/DNA of control samples was set to 100 %. Relative GAG/DNA of MC1 samples was calculated and tested against null hypothesis (*μ*0 = 100 %) using one sample *t*-test.

### Assay 4: incidence of BMSCs (CFU-F)

After red blood cell lysis of freshly harvested bone marrow aspirates, 5–10 × 10^6^ nucleated bone marrow cells were seeded on a 60 cm^2^ Petri dish in growth medium and cultured for 10–15 d. Cells were fixed and colonies were stained with 0.1 % crystal violet solution, imaged and manually counted (ImageJ v1.52p). Single measures of MC1 and control BMSCs were performed. Colony number from control bone marrow was set as 100 %. Relative MC1 colony count was calculated and tested against null hypothesis (*μ*0 = 100 %) using one sample *t*-test.

### Assay 5: bulk RNA sequencing

RNA was isolated from BMSCs harvested at 90–100 % confluency using miRNeasy Mini Kit (Qiagen) according to the manufacturer’s protocol. The library was prepared with an input of 150 ng total RNA using the TruSeq^®^ Stranded total RNA preparation kit (Illumina). Ribosomal RNA was depleted using the Ribo-Zero Gold kit (Illumina). Libraries were sequenced using the HiSeq 4000 (Illumina) sequencer (paired-end sequencing, 151 cycles, > 40 million reads per sample). The quality of data readings was checked using Fast QC. Adaptor sequences at the 3’ ends were removed and 4 bases at each end were trimmed with Trimmomatic (v0.36). Readings with > 30 nt were analysed. Readings were mapped to the reference genome hg38 (STAR; 2.6.0c), counted using FeatureCounts and statistical analysis was performed using EdgeR (v3.22.1). Genes were considered to be differentially expressed for *p* < 0.01 and Log_2_ fold change > ± 0.5. GO enrichment analysis was performed using Metacore (version 19.4) and DAVID (version 6.8) databases. GSEA was carried out with the GSEA software v4.1.0 (UC San Diego and Broad Institute, San Diego, CA, USA) (Subramanian et al., 2005) using signal to noise as gene ranking metric and 1,000 random permutations of the gene set. The analysis was performed with all hallmark gene sets from the Molecular Signatures Database (MSigDB) v7.1 (Subramanian et al., 2005) and for a specific fibrosis gene set (Wohlfahrt et al., 2019). Gene sets were considered to be enriched with FDR *q*-value < 0.1. Enrichment map visualisation was carried out in the GSEA software v4.1.0 using GSEA result of c2.cp. reactome with *p* < 0.01, FDR *q*-value < 0.1 and network was visualised in Cytoscape version 3.8.0 using an overlap coefficient of 0.5. RNA sequencing data is available at the European Nucleotide Archive (ENA) at EMBL-EBI under accession number PRJEB39993 (Web ref. 1).

### Assay 6: RT-qPCR

RNA was isolated as described above. Reverse transcription and qPCR were done according to manufacturer’s protocol (Labgene, Châtel-Saint-Denis, Switzerland). Briefly, cDNA was synthesised using the SensiFAST cDNA Synthesis Kit using 100 ng RNA. Gene expression was quantified using Mic Real-Time PCR system (Labgene) and the SensiFAST^™^ SYBR^®^ No-ROX Kit (Table 4) adding 2.5 % input of total cDNA. 40 cycles of 5 s 95 °C, 20 s 60 °C, 10 s 72 °C were run followed by melting curve analysis. Samples were measured in technical duplicates and melting curves were analysed to ensure amplification of single products. For the *COL4A5* (Hs01012435_m1), TaqMan probes were used with SensiFAST Probe No-ROX Kit. 50 cycles of 5 s 95 °C, 40 s 60 °C were run and single measures were performed. Gene expression was quantified using the ΔΔCq method with *HPRT1* as a reference gene. ΔΔCq values were tested against the null hypothesis (*μ*0 = 0) with one sample *t*-test.

### Assay 7: ELISA for pro-collagen type I alpha 1

BMSCs were grown in duplicates to 90–100 % confluency in growth medium and cultured for 48 h in starvation medium with 1 % FCS. After medium change, medium was conditioned for 24 h. A 75-fold dilution was assayed for pro-collagen type I alpha 1 using Human Pro-Collagen I alpha 1 DuoSet (R&D Systems) according to manufacturer’s protocol. Samples were measured in technical duplicates. The non-normally distributed concentrations were compared between MC1 and control using paired Wilcoxon test.

### Assay 8: collagen contraction assay

BMSCs (0.2 × 10^6^ in 1.2 mL starvation medium) were mixed with 600 μL of 3 mg/mL rat tail collagen I (Corning), 0.1 % acetic acid and 10 mmol/L NaOH. 500 μL of this mixture was added to a 24 well ultra-low attachment plate, covered with 500 μL starvation medium and incubated at 37 °C and 5 % CO_2_. Samples were measured in biological triplicates. Gels were imaged after 24 h and surface area was measured using Image J (version 1.52p). Size of MC1 BMSC gels was normalised to control BMSC gels (100 %) of the same patient tested against null hypothesis (*μ*0 = 100 %) using one sample *t*-test.

### Assay 9: Western blot

Confluent layers of BMSCs were lysed in Laemmli buffer. Proteins were separated with SDS-PAGE on 10 % polyacrylamide gels and transferred to PVDF membranes using the Trans-Blot^®^ Turbo^™^ Transfer System (BioRad). Membranes were blocked for 3 h with 5 % (w/v) non-fat dry milk (or 3 % BSA in case of p-FAK) in TBS with 0.1 % (v/v) Tween^®^ 20 and incubated overnight in 3 % (w/v) BSA in TBS with 0.1 % (v/v) Tween^®^ 20 and with primary antibodies from Cell Signaling Technologies: 1 : 1,000 rabbit anti-human β-actin (#8457), 1 : 1,000 rabbit anti-human FAK (#3285), 1 : 1,000 rabbit anti-human p-FAK (Tyr397) (#3283), 1 : 1,000 rabbit anti-human integrin β1 (#4706); or from Lucerna: 1 : 2,000 rabbit anti-human α-SMA (GTX100034). Membranes were incubated with HRP-conjugated goat anti-rabbit immunoglobulin G (IgG) (7074, Cell Signaling Technologies, 1 : 3,000) and chemiluminescence was detected using the UltraScence Pico Ultra Western Substrate (Bio-Helix, Keelung City, Taiwan) on a BioRad VersaDoc. Single measures of MC1 and control BMSCs were performed. Signal intensities of bands were determined using Image J (version 1.52a). Band intensities of α-SMA and FAK were normalised to β-actin and p-FAK was normalised to FAK. Percentages of MC1 and control intensities were compared by paired Wilcoxon test.

### Assay 10: adhesion assay

BMSC adhesion to fibronectin-coated, collagen I-coated and uncoated surface was assessed. 96-well plates were coated overnight at 4 °C using 16 μg/mL fibronectin or 0.75 mg/mL collagen I (Corning). Fibronectin- and collagen I-coated wells were blocked for non-specific binding with 1 % heat-inactivated BSA for 1 h at 37 °C. 2,500 cells/well were seeded in sextuplets. After 15 min, 30 min and 4 h, cell suspension was removed and non-adherent cells were washed away with PBS. Adherent cells were fixed using 4 % neutral buffered formalin and stained with Hoechst 33342 (ThermoFisher Scientific). 4 images per well were taken at predefined spots using a Nikon Eclipse Ti2 upright brightfield microscope. Cells were counted manually using ImageJ. Cell counts at 15 min and 30 min were normalised to the respective 4 h count. Percentage increase of adherent MC1 and control BMSCs between 15 min (settling time) and 30 min were calculated and compared using a paired *t*-test.

### Assay 11: TGF-β1 stimulation

The capacity of MC1 and control BMSCs to differentiate into myofibroblast was tested using TGF-β1 stimulation [24 h with 10 ng/mL recombinant human TGF-β1 (Peprotech)]. Myofibroblast differentiation was determined based on the expression of pro-fibrotic genes (*COL1A1*, *ACTA2*), gel contractility and *α*-SMA protein expression as described above. Effects of TGF-β1 stimulation was compared between MC1 and control using paired *t*-test.

### Assay 12: FACS of LEPR^high^ MC1 and control BMSCs

MC1 and control BMSCs were stained for LEPR as described in assay 2. The 20 % highest expressing LEPR MC1 BMSCs (LEPR^high^) were sorted using a BD FACSAria^™^ Fusion. The same sorting gate of MC1 was applied to the intra-patient control. Statistical analysis of TGF-β1 effect and adipogenic differentiation was performed as described in assay 3 and 11.

### Statistical analysis

All statistical analyses were performed using GraphPad Prism version 8.4.0. An outcome was considered significant if *p* < 0.05 (*p* < 0.01 for RNA sequencing). Normal distribution was tested using the Shapiro Wilk test. Parametric tests were run in case of normal distribution and non-parametric tests in case of non-normal distribution.

## Results

### Patient characteristics

Of the 14 included patients, 3 (21.4 %) were male and had an average age of 67.6 ± 11.6 years. Patients were in average overweight (BMI: 31.4 ± 6.3 kg/m^2^), had a median VASback of 8.0, IQR = 6.0, 9.0 and a median VASleg of 6.5, IQR = 4.5, 8.0. ODI score was high (49.3 % ± 15.7 %). Bone marrow aspirates were most often taken from vertebral level L4 (control: *n* = 5; MC1: *n* = 4) and L5 (control: *n* = 4; MC1: *n* = 6). Degree of disc degeneration (*p* = 0.098; control: 5, IQR = 3, 5; MC1: 5, IQR = 5, 5) and total endplate score (*p* = 0.011; control: 3, IQR = 1, 4; MC1: 5, IQR = 5, 6) was higher at levels of MC1 than controls.

### Stemness of MC1 BMSCs, expression of BMSC subpopulation markers and CFU-F

**Fig. 2.**
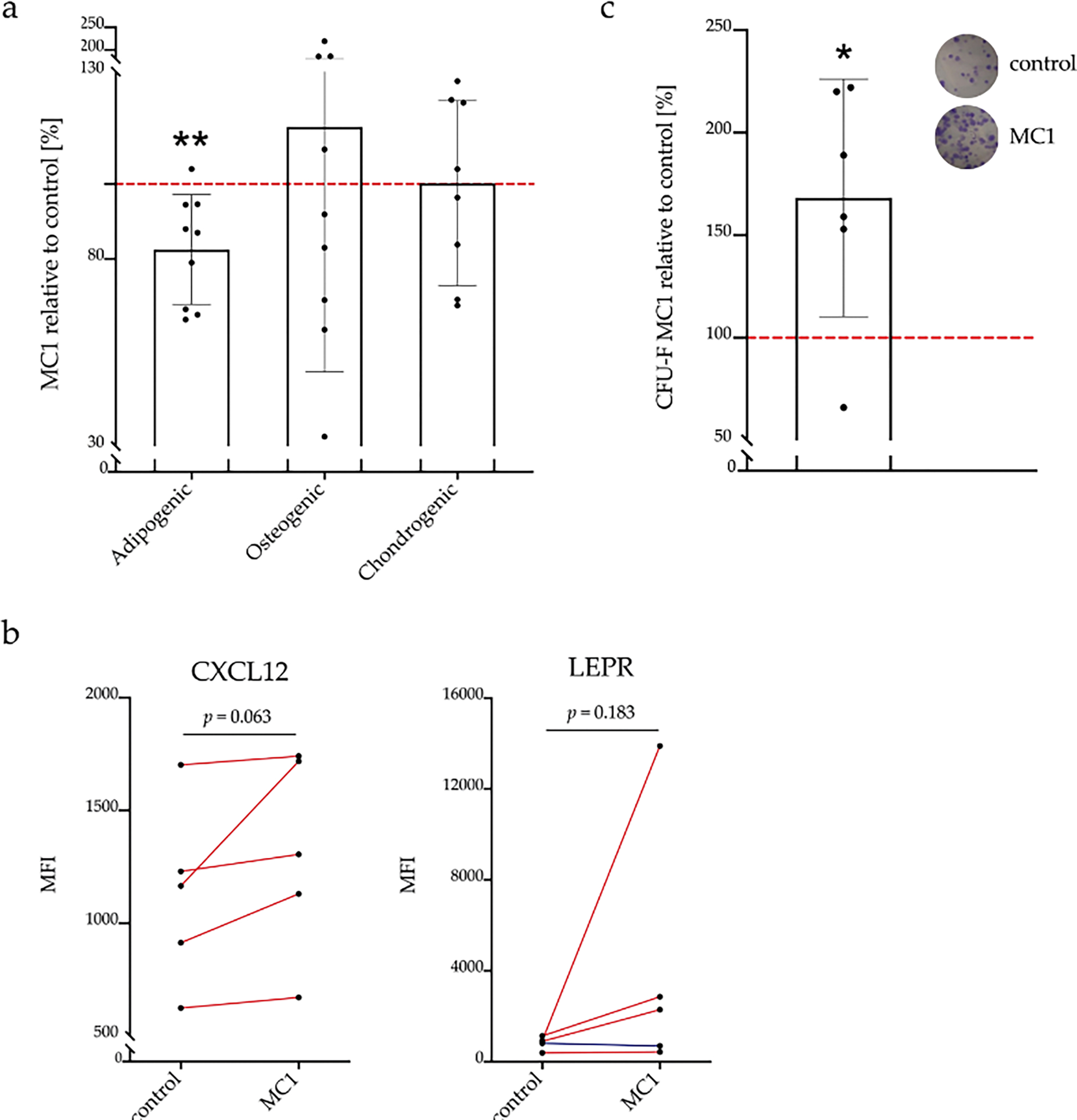
Population and stemness characterisation of MC1 BMSCs. (**a**) MC1 BMSCs had a reduced adipogenic differentiation capacity. Adipogenic, osteogenic and chondrogenic differentiation capacity of MC1 and intra-patient control was measured in sextuplicate [adipogenesis, osteogenesis (*n* = 9 + 9), chondrogenesis (*n* = 8+8)]. MC1 were normalised to intra-patient control (100 %) and tested against null hypothesis (*μ*0 = 100 %) using one sample *t*-test. (**b**) Trend towards increased expression of CXCL12 and LEPR in MC1. CXCL12 and LEPR expression was analysed by flow cytometry. Difference in MC1 to control ΔMFI was tested against null hypothesis (***μ***0 = 0) using Wilcoxon test. Single measures of MC1 and control were performed (*n* = 5 + 5). (**c**) Increased incidence of BMSCs in MC1. CFU-F from MC1 and control bone marrow mononuclear cells were fixed, stained and manually counted using Image J. Colony number from control bone marrow was set as 100 %. Relative MC1 colony count was calculated and tested against null hypothesis (*μ*0 = 100 %) using one sample *t*-test (*n* = 6 + 6). Single measures of MC1 and control were performed. *p < 0.05, **p < 0.01.

MSCs are defined by their expression of the consensus surface markers and their ability to differentiate into adipocytes, osteoblasts and chondrocytes. BMSCs from control and MC1 both expressed high levels of the consensus marker sequence without significant differences (*p* = 0.527; MC1: 93.2 % ± 4.6 %; control: 93.8 % ± 3.7 %). A decreased adipogenic differentiation potential of MC1 BMSCs compared to intra-patient control BMSCs (− 17.5 % ± 14.7 %, *p* = 0.007) was observed. There was no significant difference in osteoblast (+ 15.2 % ± 65.4 %, *p* = 0.505) and chondrocyte differentiation capacity (− 0.9 % ± 24.0 %, *p* = 0.915) (Fig. 2a).

Flow cytometric analysis of markers describing BMSC subpopulations revealed no significant changes in expression of all tested surface markers (LEPR, CXCL12, CD54, CD140a, CD146, CD271, NG2, CD106, nestin) (Table 5). Despite the high variability, in all tested patients, CXCL12 was higher expressed in MC1 than control (*p* = 0.063) and LEPR was higher expressed in 4 out of 5 MC1 cells (*p* = 0.180) (Fig. 2b). Expression of LEPR and CXLC12 correlated (*r* = 0.95, *p* = 0.014). Furthermore, expression of CD271, NG2 and CD106 correlated (*r* = 0.91–0.99, *p* = 0.001–0.031) as well as nestin and CD146 (*r* = 0.99, *p* = 0.002).

To quantify the relative frequency of BMSCs in MC1 bone marrow, CFU-F of nucleated cells was quantified in MC1 and control bone marrow aspirates. MC1 contained more CFU-F than control bone marrow (+ 68.2 % ± 57.9 %, *p* = 0.035) (Fig. 2c).

### RNA sequencing and expression of pro-fibrotic genes and proteins

**Fig. 3.**
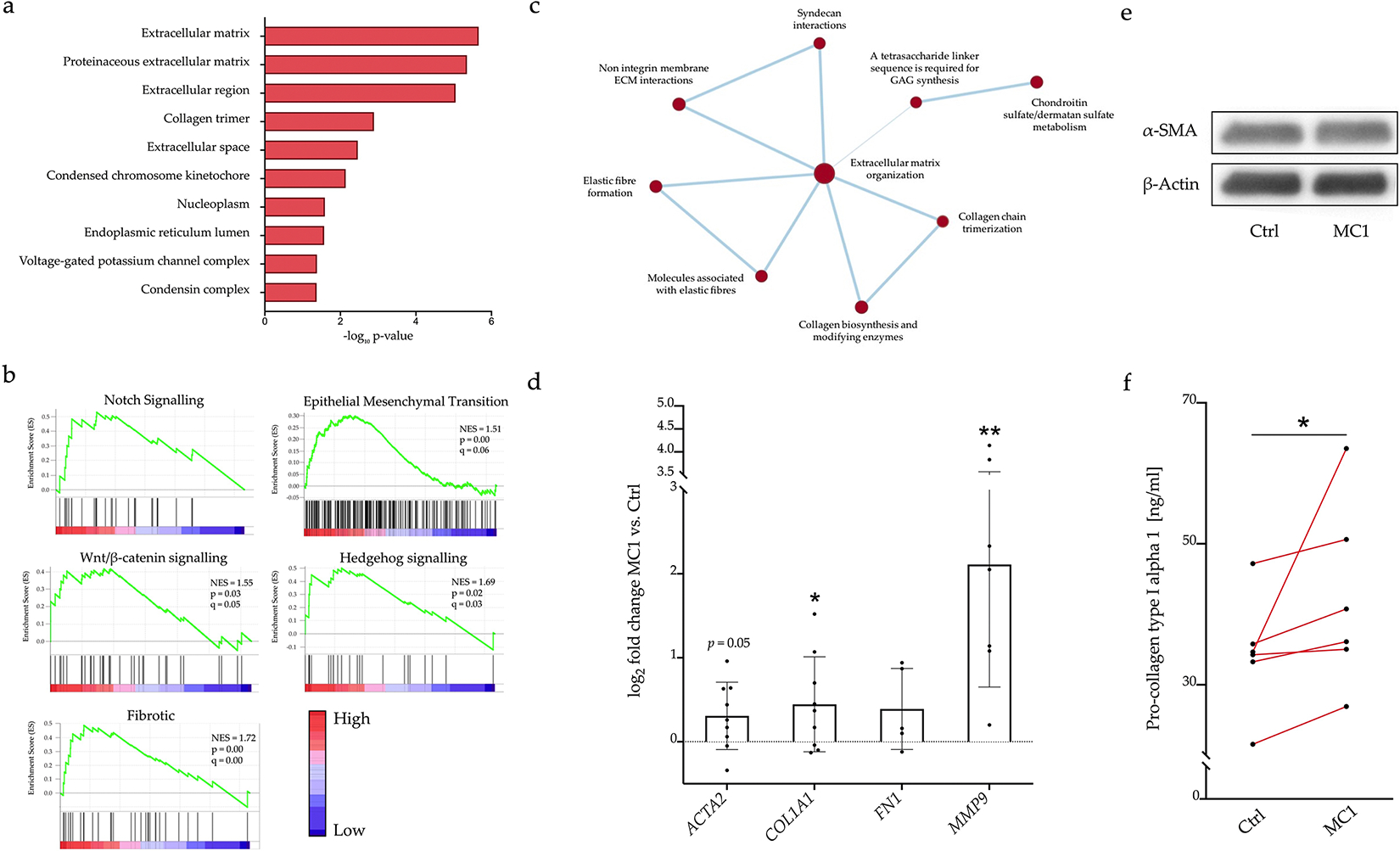
Transcriptome profile of MC1 *versus* intra-patient control BMSCs. (**a-c**) Bulk RNA sequencing of *in vitro*-expanded MC1 and intra-patient control BMSCs (*n* = 4 + 4) was performed (Illumina). Genes were considered as DEG for *p* < 0.01 and Log_2_ fold change > ± 0.5. GO enrichment was performed in DAVID, GSEA with GSEA software. (**a**) The 10 most significantly overrepresented cellular components comparing MC1 to control. (**b**) GSEA of “hallmark” gene sets revealed enriched pro-fibrotic biological processes. (**c**) Enrichment map visualisation of pathways (Reactome database) enriched in MC1 BMSCs with gene sets *p* < 0.01, FDR *q*-value < 0.1 and an overlap coefficient of 0.5. Node size displays number of genes within a gene set, thickness of connection line represents extent of overlap between gene sets. (**d**) Expression of pro-fibrotic genes was up-regulated in MC1 BMSCs. Pro-fibrotic gene expression was compared between MC1 and control by RT-qPCR. Samples were measured in technical duplicates (*n* = 5 + 5 – 9 + 9) and ΔΔCq (MC1 to control) values were tested against the null hypothesis (*μ*0 = 0) with one sample *t*-test. Log_2_ fold change of ΔΔCq are represented. (**e**) Representative Western blot of α-SMA expression. α-SMA protein level was detected by Western blot (*n* = 10 + 10) and band signal intensities were quantified 3 times using Image J. α-SMA band signal intensities were normalised to β-actin and percentages of MC1 and control intensities were compared with paired Wilcoxon test. (**f**) Synthesis of pro-collagen type I alpha 1 was increased in MC1 BMSCs. BMSCs were cultured for 48 h in starvation medium with 1 % FCS. After medium change, medium was conditioned for 24 h and pro-collagen type I alpha 1 was measured by ELISA. MC1 and control were measured in biological duplicates and ELISA was performed in technical duplicates. *p < 0.05; **p < 0.01.

Bulk RNA sequencing comparing MC1 to control BMSCs identified 219 DEGs. GO analysis of the DEGs with DAVID (*p* = 2.2 × 10^− 6^) and Metacore (*p* = 7.2 × 10^− 13^) revealed an enrichment of the ECM (Table 6). In the GO class “cellular components”, the top items were all related to ECM or collagen and were all up-regulated (Fig. 3a). “Extracellular matrix structural constituent” (*p* = 2.3 × 10^− 6^) and “extracellular matrix organisation” (*p* = 3.5 × 10^− 4^) were also the top enriched GOs in the classes “molecular function” and “biological process”, respectively. GSEA identified the Notch pathway, Wnt/β-catenin pathway, Hedgehog pathway and EMT as top enriched gene sets. Furthermore, a specific fibrosis gene set was highly enriched (Fig. 3b). GSEA of the canonical pathways gene sets derived from the Reactome pathway database (820 gene sets) and enrichment map visualisation in Cytoscape identified “extracellular matrix organisation” as a central process connected with several functions related to ECM formation and modification (Fig. 3c).

Analysis of pro-fibrotic gene expression by qPCR revealed a slight upregulation of *ACTA2* (Log_2_ fold-change = 0.31 ± 0.40, *p* = 0.050) and of *COL1A1* (Log_2_ fold-change = 0.45 ± 0.57, *p* = 0.046) as well as a marked up-regulation of *MMP9* (Log_2_ fold-change = 2.11 ± 1.46, *p* = 0.0009) in MC1 (Fig. 3d). The increased *ACTA2* expression in MC1 could not be confirmed on the protein level as measured by Western blot (*p* = 0.922, − 2.24 %, IQR = − 25.09 %, + 22.4 %) or flow cytometry (*p* = 0.467, + 17.1 ± 47.6 %) (Fig. 3e). Concentration of pro-collagen type I alpha 1 was significantly higher in starvation medium from MC1 BMSCs compared to control BMSCs (*p* = 0.031, + 11.2 %, IQR = + 7.60 %, 22.07 %) (Fig. 3f).

### Gel contraction, matrix adhesion and expression of FAK/p-FAK

**Fig. 4.**
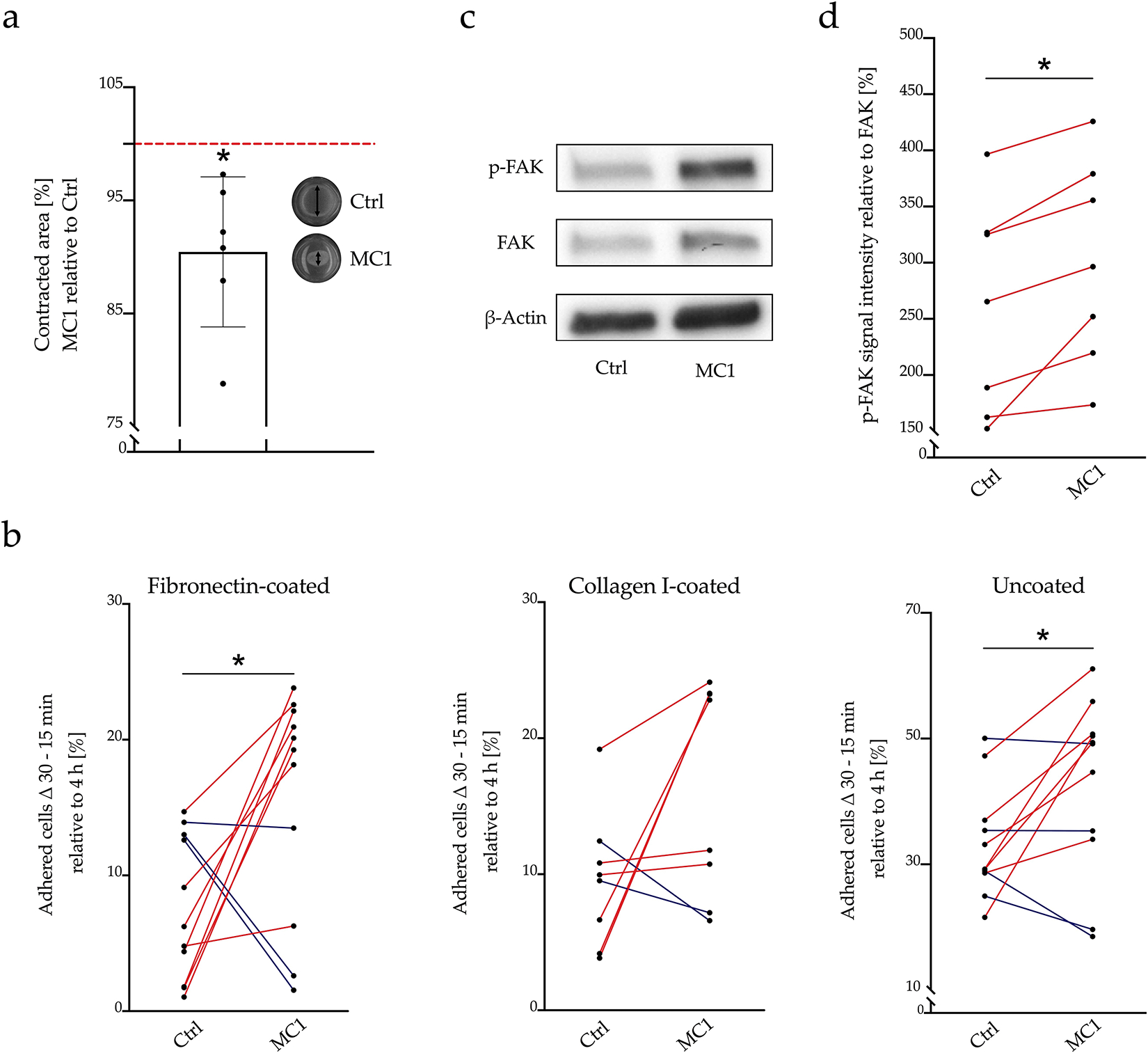
Collagen gel contractility and cell matrix adhesion. (**a**) MC1 BMSCs contracted gels more than the respective control BMSCs. MC1 and control collagen gel contractility was measured in biological duplicates. BMSCs were mixed with rat tail collagen I and incubated for 24 h. Gel surface area was measured using Image J. Size of MC1 BMSC gels was normalised to control BMSC gels (100 %) of the same patient and tested against null hypothesis (*μ*0 = 100 %) using one sample *t*-test. (**b**) Cell-matrix adhesion: increased adhesion of MC1 BMSCs to fibronectin and uncoated plates. BMSC adhesion to fibronectin-coated, collagen I-coated and uncoated (*n* = 8 + 8 – 11 + 11) plates were assessed between 15 and 30 min after cell seeding by counting adhered cells. Cell numbers were normalised to the respective 4 h counts. Percentage increase of adherent MC1 and control BMSCs were compared by paired *t*-tests. MC1 and control adhesion was measured in sextuplicate. (**c**) p-FAK was increased in MC1 BMSCs. FAK and p-FAK protein level were detected by Western blot (*n* = 7 + 7) and band signal intensities were quantified 3 times using Image J. p-FAK band signal intensities were normalised to total FAK (% total FAK) and percentages of MC1 and control intensities were compared with paired Wilcoxon test. (**d**) Representative Western blot of p-FAK, FAK and β-actin. *p < 0.05.

Contraction capacities of BMSCs were quantified by their ability to contract collagen gels. MC1 BMSCs contracted collagen gels more than control BMSCs, resulting in smaller gels after 24 h (− 9.6 % ± 6.62 %, *p* = 0.017) (Fig. 4a).

Adhesion to matrix of MC1 and control BMSCs was compared by counting the cells that attached to fibronectin-coated, collagen I-coated or uncoated dishes. More cells bound to fibronectin-coated dishes (+ 7.9 % ± 11.6 %, *p* = 0.047) and to uncoated dishes (+ 9.4 % ± 12.9, *p* = 0.036). The increase in binding to type I collagen-coated dishes was not significant (+ 6.6 % ± 10.1, *p* = 0.110) (Fig. 4b).

Whether the increased matrix adhesion was due to increased expression of integrins was analysed. There was no difference in the gene expression of all tested integrins except for *ITGB1* (Log_2_ fold-change = 0.32 ± 0.26, *p* = 0.022) (Table 7). However, flow cytometric analysis (*p* = 0.871) and Western blot of ITGB1 (*p* = 0.938, + 5.31 %, IQR = − 25.9 %, + 12.7 %) showed no significant difference in ITGB1 protein level (data not shown).

Phosphorylation of FAK is central in downstream signalling of integrins and is important in mediating cell contractility (Vallée and Lecarpentier, 2019). Therefore, the fraction of phosphorylated FAK to total FAK was measured and calculated by Western blot. The percentage of phosphorylated FAK from total FAK (p-FAK/FAK) was significantly higher in MC1 BMSCs (+ 30.9 %, IQR = + 29.8 %, 40.7 %, *p* = 0.016) while total FAK normalised to β-actin (FAK/β-actin) was not increased (+ 2.8 %, IQR = − 1.1 %, 8.7 %, *p* = 0.468) (Fig. 4c,d).

### Responsiveness to TGF-β1 of bulk BMSCs

**Fig. 5.**
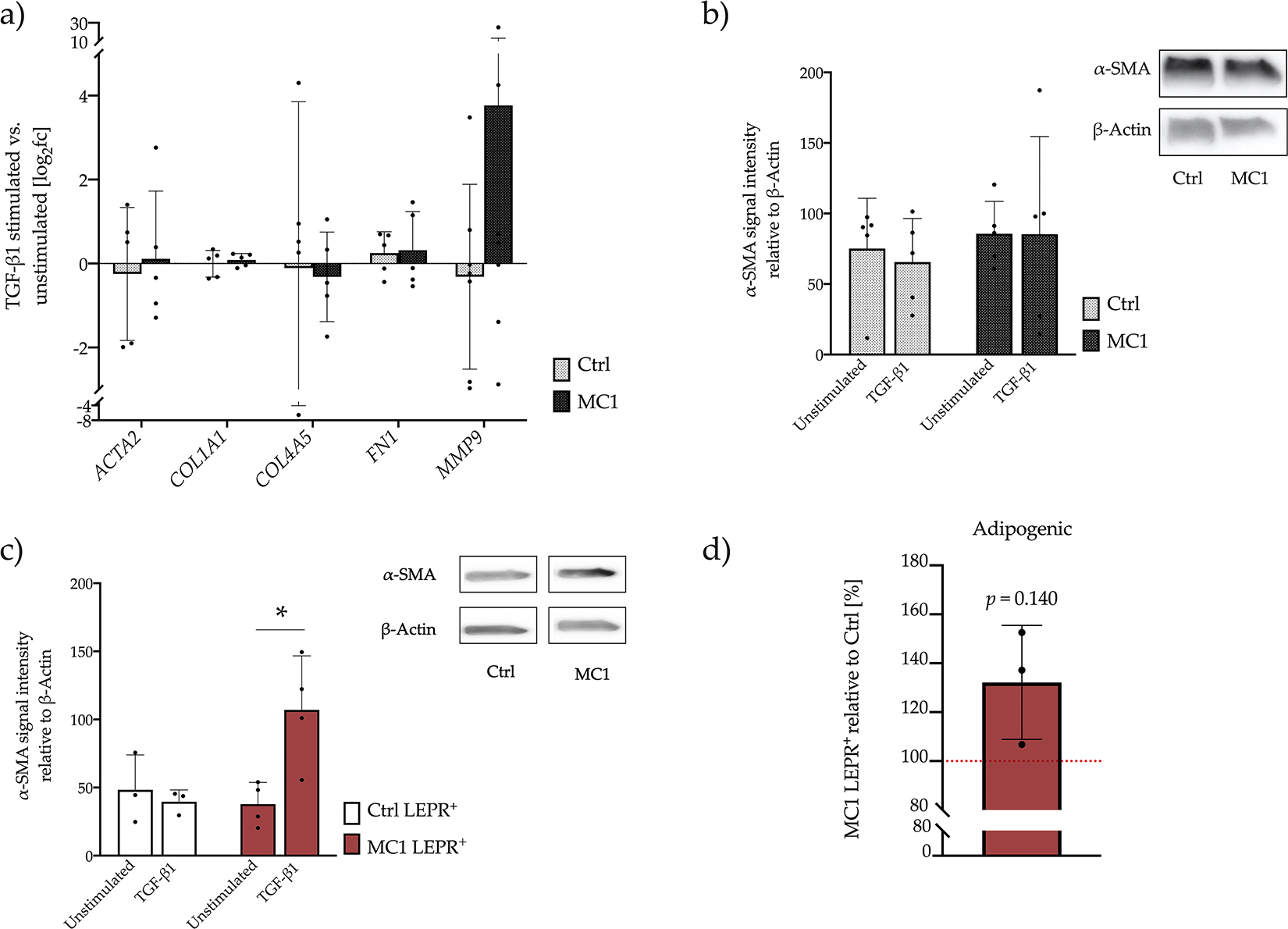
MC1 and control BMSCs in response to TGF-β1. (**a**) TGF-β1 stimulation did not result in a different pro-fibrotic gene expression between MC1 and control BMSCs. Gene expression of 24 h stimulation with 10 ng/mL TGF-β1 was measured by RT-qPCR (*n* = 5 + 5) in technical duplicates. Effects of TGF-β1 stimulation was compared between MC1 and control by paired *t*-test. (**b**) No difference in α-SMA protein level upon TGF-β1 stimulation in MC1 and control BMSCs. α-SMA protein level of TGF-β1-stimulated and -unstimulated MC1 and control BMSCs was detected by Western blot (*n* = 5 + 5). Band signal intensities were quantified 3 times using Image J. Effects of TGF-β1 stimulation were compared by paired *t*-test. (**c**) TGF-β1 stimulation of LEPR^high^-sorted MC1 BMSCs resulted in a significant increase in α-SMA production, whereas there was no change in LEPR^high^-sorted control BMSCs. α-SMA protein level in TGF-β1-stimulated and -unstimulated LEPR^high^-sorted MC1 (*n* = 4) and control BMSCs (*n* = 3) was detected by Western blot. Band signal intensities were quantified 3 times using Image J. Effects of TGF-β1 stimulation were compared by paired *t*-test. (**d**) LEPR^high^ MC1 BMSCs had a trend towards increased adipogenic differentiation capacity. Adipogenic differentiation capacity of LEPR^high^-sorted MC1 and intra-patient control was measured in sextuplicate (*n* = 3 + 3). LEPR^high^ MC1 was normalised to intra-patient LEPR^high^ control (100 %) and tested against null hypothesis (μ0 = 100 %) using one sample *t*-test. Log_2_fc: Log_2_ fold-change.*p < 0.05.

The pro-fibrotic gene expression of MC1 and control BMSCs in response to 24 h TGF-β1 stimulation was compared. qPCR analysis revealed high interpatient variability and no increase in *ACTA2* expression after stimulation in MC1 (Log_2_ fold-change = 0.11 ± 1.61) and control (Log_2_ fold-change = − 0.25 ± 1.58) and no difference between MC1 and control (*p* = 0.648). Similarly, *COL1A1* (Log_2_ fold-change MC1 = 0.09 ± 0.15, Log_2_ fold-change control = − 0.01 ± 0.32, *p* = 0.656), *COL4A5* (Log_2_ fold-change MC1 = 0.31 ± 0.93, Log_2_ fold-change control = − 0.11 ± 3.97, *p* = 0.920), *FN1* (Log_2_ fold-change MC1 = 0.32 ± 0.92, Log_2_ fold-change control = 0.25 ± 0.51, *p* = 0.900) and *MMP9* (Log_2_ fold-change MC1 = 3.77 ± 8.98, Log_2_ fold-change control = − 0.31 ± 2.04, *p* = 0.302) expression remained unchanged (Fig. 5a). α-SMA expression measured by Western blot upon TGF-β1 stimulation was not significantly increased in MC1 (− 0.3 % ± 75.71 %) and control (− 9.54 % ± 48.01 %) and did also not differ between MC1 and control BMSCs (*p* = 0.606) (Fig. 5b). Additionally, there was no difference in collagen gel contraction in response to TGF-β1 in MC1 (− 2.39% ± 15.62 %) and control (+ 12.57 % ± 14.52 %) and there was also no difference between MC1 and control BMSCs (*p* = 0.210).

### Analysis of LEPR^high^ BMSCs

To test if LEPR^high^ BMSCs could undergo myofibroblast differentiation, α-SMA was quantified by Western blot after TGF-β1 stimulation of sorted LEPR^high^ BMSCs of MC1 and control. Whereas α-SMA expression upon TGF-β1 stimulation in LEPR^high^ control BMSCs was not changed (− 8.7 % ± 33.55 %, *p* = 0.693), TGF-β1 stimulation led to a strong increase in α-SMA expression in LEPR^high^ MC1 BMSCs (+ 69.23 % ± 37.9 %, *p* = 0.035) (Fig. 5c). Furthermore, the adipogenic differentiation capacity of LEPR^high^ sorted BMSCs was compared. LEPR^high^ MC1 BMSCs of the 3 analysed patients had a trend towards an increased adipogenic differentiation capacity (+ 32.17 % ± 23.29 %, *p* = 0.140) (Fig. 5d).

## Discussion

Bone marrow fibrosis is a pathophysiological important mechanism in MC1 and may have diagnostic and clinical relevance (Dudli et al., 2017; Dudli et al., 2020; Modic et al., 1988). The present study showed that BMSCs in MC1 have a pro-fibrotic phenotype compared to BMSCs from control vertebral bone marrow from the same patient. This phenotype is characterised by excessive production of ECM, increased cell contraction capability and enhanced adhesion. Increased FAK phosphorylation was identified as an important fibrotic characteristic of MC1 BMSCs, suggesting a mechanistical link between increased cell adhesion and cell contractility. An overrepresented LEPR^high^ subpopulation in MC1 but not control could undergo myofibroblast differentiation and, hence, be a possible driver of fibrosis in MC1.

### Increased CFU-F and LEPR expression and reduced adipogenic differentiation capacity in MC1

BMSCs are a heterogeneous population of fibroblastic cells. Sub-populations of BMSCs are responsible for bone marrow fibrosis in other conditions (Decker et al., 2017; Schneider et al., 2017). Whether the BMSC population in MC1 was different from the control BMSC population of the adjacent vertebra was investigated. CD14^−^, CD19^−^, CD34^−^, CD45^−^, CD73^+^, CD90^+^ and CD105^+^ marker expression was compared. Even though these markers are indistinguishably expressed on fibroblasts and MSCs, this surface marker expression combination is an important criterion that defines MSCs (Denu et al., 2016; Dominici et al., 2006). MC1 and control BMSCs did not differ in the expression of the MSC consensus markers CD14^−^, CD19^−^, CD34^−^, CD45^−^, CD73^+^, CD90^+^ and CD105^+^ but MC1 BMSCs tended to express more CXCL12 and LEPR with correlating expression of CXCL12 and LEPR. Despite the high patient variability in absolute expression levels, these findings were consistent and may indicate that a CXCL12^+^ LEPR^+^ population was overrepresented in MC1. CXCL12^+^ LEPR^+^ cells are a perisinusoidal population that create a niche for haematopoietic stem cells and give rise to osteoblasts (Acar et al., 2015; Galán-Díez and Kousteni, 2018). Neovascularisation in MC1 may go along with expansion of a CXCL12^+^ LEPR^+^ population on the newly formed sinusoidal surface (Bailey et al., 2011). Increased number of CFU-F in MC1 could support this notion as almost all CFU-F are LEPR^+^ BMSCs (Decker et al., 2017; Zhou et al., 2014). Expansion of CXCL12^+^ LEPR^+^ cells could be a response to endplate and trabecular bone damage in MC1 because LEPR^+^ BMSCs proliferate after bone injury and give rise to osteoblasts (Galán-Díez and Kousteni, 2018; Zhou et al., 2014). Importantly, CXCL12^+^ LEPR^+^ cells have been identified as a major source of myofibroblasts in bone marrow fibrosis in primary myelofibrosis (Decker et al., 2017). Although primary myelofibrosis is a distinct pathological entity, the study by Decker et al. (2017) showed that CXCL12^+^ LEPR^+^ cells are capable of undergoing myofibroblast differentiation and contribute to bone marrow fibrosis.

LEPR-expressing BMSCs are the main progenitor cells that generate adipocytes, osteoblasts and chondrocytes in the bone marrow (Zhou et al., 2014). Differentiation of BMSCs in MC1 may be affected by the inflammatory environment (Huang et al., 2014), bony endplate damages (Zhou et al., 2014) and pro-osteoclastic/anti-osteoblastic factors draining from the adjacent intervertebral discs through endplate damages into the bone marrow (Dudli et al., 2017; Rajasekaran et al., 2004; Torkki et al., 2016). In this complex environment, a reduced adipogenic differentiation capacity but no significant difference in osteogenic and chondrogenic differentiation were found. This indicated a net loss of the trilineage differentiation capacity and stemness and suggested an unspecific fibroblastic polarisation of MC1 BMSCs. A loss of adipogenic differentiation capacity of fibroblasts has also been shown in a bleomycin-induced mouse model of fibrosis (El Agha et al., 2017b). Lung fibroblasts underwent a shift away from adipogenic towards myofibroblasts polarisation, while resolution of fibrosis reversed the shift. However, the present study results indicated that LEPR^high^ BMSCs did not contribute to the reduced adipogenic differentiation capacity. In contrast, LEPR^high^ BMSCs in MC1 tended to differentiate more into adipocytes than LEPR^high^ BMSCs of controls. This corresponded with the finding of Yue et al. (2016) who showed that leptin receptor on mesenchymal stromal cells promotes adipogenesis. The present study findings suggested that the reduced adipogenic differentiation capacity of “bulk” MC1 BMSCs was caused by the LEPR^low^ BMSC population and that a myofibroblastic BMSC phenotype was rather linked to increased adipogenesis than reduced adipogenesis. The underlying mechanisms for the altered adipogenic differentiation capacities in LEPR^high^ and LEPR^low^ remain unknown.

Together, these findings suggested that a perivascular population was overrepresented in MC1 and that MC1 BMSC differentiation capacity was reduced. The reduced adipogenic differentiation capacity could most likely not be attributed to the overrepresented LEPR^high^ population in MC1 BMSCs. It needs to be shown if the over represented population and the reduced adipogenic differentiation potential of MC1 BMSCs were causally linked to fibrosis in MC1.

### Pro-fibrotic ECM production and signalling in MC1 BMSCs

The transcriptome of MC1 BMSCs showed a pro-fibrotic phenotype of MC1 BMSCs, with increased ECM deposition and activation of pro-fibrotic signalling pathways. ECM constituents and modifying enzymes were enriched in GO analysis and were the most enriched gene sets in the pathway analysis. Increased synthesis of ECM is a hallmark of fibrosis and is causally linked to clinical manifestations in many fibrotic pathologies as it restricts proper tissue function (Wynn, 2008). Synthesis of pro-collagen type I alpha 1, the prototypical ECM constituent secreted by myofibroblasts, was increased in MC1 BMSCs and type XXII collagen alpha 1 and type IV collagen alpha 5 were the top dysregulated ECM constituents of the MC1 BMSC transcriptome. Type XXII collagen has been identified as a marker of the transition of skin fibroblasts to myofibroblasts in systemic sclerosis (Watanabe et al., 2019). Type IV collagen is a prototypic ECM protein of myofibroblasts and pro-collagen type IV turnover has been identified as a prognostic marker for the disease progression of systemic sclerosis [Dobrota et al. (2020). Circulating collagen turnover markers are specifically changed in very early systemic sclerosis. Ann Rheum Dis 79: 159; conference abstract; Klingberg et al., 2013]. Importantly, circulating pro-collagen type IV has been reported to be increased in peripheral blood of MC1 patients (Dudli et al., 2020). This shows that increased ECM secretion through fibrotic mechanisms in MC1 has a potential diagnostic value. Findings suggested a link between increased serum values of type IV collagen and the fibrotic pathomechanism in MC1 bone marrow. If this can be validated, it is an important finding as it provides face validity for circulating pro-collagen-4 as MC1 biomarker and underscores the clinical significance of bone marrow fibrosis in MC1.

Besides increased ECM secretion, fibrosis and EMT were identified, as well as the three signalling pathways, Notch, Wnt/β-catenin and Hedgehog as the top enriched gene sets in MC1. This corroborated the pro-fibrotic phenotype of MC1 BMSCs as all three signalling pathways mediated fibrotic processes across different organs and tissues, including lung, kidney, skin, liver and heart (Burgy and Königshoff, 2018; Hu et al., 2015; Hu and Phan, 2016; Lam and Gottardi, 2011). Inhibition of Notch, Wnt/β-catenin and Hedgehog signalling inhibits tissue fibrosis through mechanisms that inhibit EMT (Fontaine et al., 2008; Wang et al., 2019; Yuan et al., 2016). Notch signalling also promotes PMT, which could explain the increase in the pericyte-like CXCL12^+^ LEPR^+^ population. Furthermore, Notch, Wnt/β-catenin and Hedgehog signalling affect MSC differentiation. Notch signalling generally inhibits any lineage differentiation but enhances MSC proliferation (Dong et al., 2010). Hedgehog and Wnt/β-catenin signalling promote osteoblast differentiation and inhibit adipocyte differentiation (Fontaine et al., 2008; Yuan et al., 2016). Therefore, increased Notch, Wnt/β-catenin and Hedgehog signalling may relate to increased CFU-F and decreased adipogenic differentiation capacity as found in the present study.

### Increased cell adhesion, cell contractility and FAK phosphorylation

Phosphorylation of FAK has been identified as an important mechanism in fibroblasts causing bone marrow fibrosis and lung fibrosis (Desterke et al., 2015; Lagares et al., 2012; Zhao et al., 2016). Increased matrix adhesion, gel contractility and FAK phosphorylation were found in MC1 BMSCs. This was a strong indication for a pro-fibrotic phenotype of MC1 BMSCs and that FAK was important in MC1 fibrosis.

FAK is a key kinase of the ECM-cytoskeleton mechanotransduction *via* integrins and mediates several fibrotic mechanisms. FAK modulates adhesion strength through integrin activation and modulates cell contractility by α-SMA and actin polymerisation at focal adhesions (Michael et al., 2009). Phosphorylation of FAK occurs after integrin binding to matrix and is associated with the cytoplasmic domain of integrin β subunits. Integrins are αβ-heterodimers and, of the β-subunits, integrin β1 conveys a key role as it binds almost all α-subunits to form matrix receptors. The α-subunit defines the specificity of the integrin receptor to bind fibronectin, collagen or laminin. In fibrosis, integrin β1 plays a central role and its inhibition or knock-down reduces fibrosis (Basta et al., 2020; Liu et al., 2009; Zhao et al., 2016). MC1 BMSCs adhered stronger to fibronectin and uncoated plastic dishes, indicating an increased non-specific matrix binding. Next, it was assessed whether increased non-specific binding was caused by overexpression of specific integrins and in particular of the universal subunit integrin β1. No change in gene transcription of 16 tested integrin subunits was found, except for integrin β1, which was marginally but significantly upregulated. However, increased integrin β1 expression could not be validated at protein level by Western blot and flow cytometric analysis. Matrix adhesion is also modulated by FAK through ‘inside-out’ signalling that activates integrins through conformational changes (Michael et al., 2009). Therefore, increased matrix adhesion in MC1 BMSCs was not due to overexpression of integrins but might be due to FAK phosphorylation through other pathways.

Phosphorylation of FAK is important for the formation of α-SMA stress fibres that mediate cell contractility. MC1 BMSCs had an increased cell contractility without a relevant increase in α-SMA expression. This suggested a mechanism independent of α-SMA expression. Organisation and polymerisation of actin is FAK-mediated and an important early step in the formation of focal adhesion that transduce mechanical forces. It might be that MC1 BMSCs had a more efficient way to form nascent adhesions and increase cell contractility without increasing α-SMA expression.

### Responsiveness to myofibroblast differentiation

Myofibroblasts are α-SMA-expressing highly contractile cells that produce large amounts of ECM. TGF-β1 is a master regulator of fibrosis and promotes myofibroblast differentiation (Van Caam et al., 2018). No difference in pro-fibrotic gene expression, α-SMA production and collagen gel contraction were found between MC1 and control BMSCs in response to TGF-β1. As Decker et al. (2017) identified LEPR-expressing BMSCs as the cellular origin of the collagen-producing myofibroblasts in bone marrow fibrosis, it was investigated whether TGF-β1 induced myofibroblast differentiation of LEPR^high^ BMSCs. Interestingly, LEPR^high^-sorted MC1 BMSCs had a significantly upregulated α-SMA production upon TGF-β1 stimulation, whereas LEPR^high^-sorted control BMSCs had not. These results showed two things. First, that a LEPR^high^ subpopulation of MC1 BMSCs could undergo myofibroblast differentiation, which corresponds to the bone marrow fibrosis contributing CXCL12^+^ LEPR^+^ phenotype discovered by Decker et al. (2017). Second, since LEPR^high^ control BMSCs did not undergo TGF-β1-induced myofibroblast differentiation, LEPR expression on BMSCs was not a sufficient criterion for myofibroblast differentiation. Therefore, MC1 LEPR^high^ BMSCs but not control LEPR^high^ BMSCs were susceptible to differentiate into myofibroblasts. It remains unknown what makes MC1 LEPR^high^ BMSCs susceptible to myofibroblast differentiation, yet chronic inflammation as well as biomechanical changes within MC1 bone marrow might be critical factors.

In summary, while collectively BMSCs in MC1 had a pro-fibrotic phenotype, only a LEPR^high^ BMSC population in MC1 could undergo myofibroblast differentiation. The role of the different BMSC subpopulations in the fibrotic pathomechanism of MC1 awaits investigations. It also needs to be demonstrated if MC1 bone marrow indeed contains α-SMA positive myofibroblasts.

### Limitations and critical evaluation

BMSCs from patients with lumbar MC1 who underwent lumbar spinal fusion were investigated. Since LBP is a multifactorial disease and MC1 is not an indication for surgery, patients had different surgical indications. Most suffered from spinal stenosis, listhesis, scoliosis, sagittal misalignment or facet joint degeneration at the surgical level. Hence, different aetiologies may have caused MC1. However, mechanical overload and endplate damage always occur in MC1 and seem to be independent of the primary diagnosis (Dudli et al., 2016). Therefore, pathophysiological bone marrow changes in MC1 may be less diverse than their aetiology. Nevertheless, to select patients with mainly axial and not radicular pain, only patients with higher VAS scores for back pain than leg pain were enrolled. Also, patients with prior spinal fusion, malignancies and infectious disease were excluded to eliminate potential confounders.

Size and type of MC1 (pure MC1 *vs.* mixed MC1/MC2) may also affect BMSC phenotype. Only BMSCs from MC lesions that were classified as MC1 or mixed MC1/MC2 were investigated. Pure MC1 are rare, in particular in patients undergoing spinal fusion, as this is the treatment of last resort and MC1 at the time of operation may have evolved over time through different phases of MC1 and MC2 before.

The quality of the aspirate may affect the investigated BMSC population. Intraoperative X-ray confirmed that the bone marrow aspiration needle was placed at the correct site before taking the aspirate. However, it was not possible to monitor the marrow space which was aspirated. Therefore, it was not possible to validate post-operatively if MC1 aspirate contained cells only from MC1 lesions or if cells from non-affected areas were aspirated as well. Control bone marrow is heterogeneous as well and often shows small areas of signal intensity changes related to focal changes in marrow composition. Therefore, aspirates from control marrow may have also contained some potential pathological cells. The aspiration volume was limited to maximal 3.5 mL. This volume limits the risk of aspirating peripheral blood and bone marrow cells outside the target area (Brooimans et al., 2009) and at the same time guarantees a large enough cell pool to obtain a representative population from the target area.

Culturing BMSCs changes their expression profile (Ghazanfari et al., 2017). However, a thorough characterisation of primary BMSCs is not possible because primary BMSCs are a rare population in bone marrow and not enough cells would have been available for all assays. MC1 are a chronic condition and many changes are likely stably imprinted and do not change during expansion. Therefore, cells from MC1 and control were expanded *in vitro* and always used at the same passage (passage 3, 4 or 5) to minimise any culture expansion effects.

It was not possible to correlate changes across different assays as not all were performed with cells from the same patient. This limits a more powerful interpretation of the data as patient-to-patient variation was considerable for most assays. However, MC1 BMSCs were always compared to intra-patient control BMSCs for each assay, which eliminated most of patient-related confounders and allowed for a paired analysis.

## Conclusion

In summary, the present is the first study characterising BMSCs in MC1. Important pro-fibrotic changes in MC1 BMSC and a LEPR^high^ MC1 BMSC subpopulation susceptible to myofibroblast differentiation were found. Fibrosis is a hallmark of MC1 and BMSCs in MC1 represent a potential therapeutic target to control fibrosis in MC1. A causal link between the pro-fibrotic phenotype and clinical characteristics needs to be demonstrated.

## Figures and Tables

**Table 1.** Patient demographics.

ID	Age	Male (m)/ female(f)	Height [cm]	Weight [kg]	BMI [kg/m^2]	Smoker	VASback	VASleg	ODI [%]	Ctrl	MC1	DD Ctrl	DD MC1	TES Ctrl	TES MC1		Assays

1	70	f	161	91	35.1	yes	9	9	66	L4	L5	5	5	4	5	< 50 %	3,5,6,7,8,9,10
2	87	f	156	76.6	31.5	no	9	0	66	L2	L5	5	5	6	6	> 50 %	1,3,5,6,7,9,10,11
3	73	f	155	91.5	38.1	no	NA	NA	36	L4	S1	5	5	1	4	< 50 %	3,5,6,7,9,10,11
4	80	m	170	75	26.0	no	9	8	48	L3	L4	5	5	3	5	< 50 %	3,5,6,7,11
5	58	f	151	68.7	30.1	yes	6	NA	NA	L4	L5	NA	NA	NA	NA	NA	1,3,4,5,7,8,9,10,11
6	68	m	188	166.9	47.2	no	8	6	52	S1	L5	5	5	3	6	< 50 %	1,2,3,4,5,7,9,10,11
7	58	f	153	90	38.4	yes	8	8	53	L5	S1	1	5	1	6	< 50 %	1,3,4,5,7,9,10,11
8	55	m	182	106.3	32.1	yes	6	6	30	L5	L3	5	5	6	4	> 50 %	1,3,4,7,9,10,11
9	75	f	156	84	34.5	no	8	0	25	L3	L4	5	5	3	5	> 50 %	1,3,4,7,8,10,11
10	73	f	162	79.5	30.3	no	0	7	51	L2	L4	2	5	1	6	> 50 %	2,7,8,9,10,11
11	50	f	174	105	34.7	no	9	9	82	L4	L5	3	5	1	5	> 50 %	2,8,9,10,11
12	55	f	172	60	20.3	yes	7	8	50	L5	L4	5	5	3	3	< 50 %	2,8,9,10,11
13	83	f	163	69	26.0	yes	7	5	38	L4	L5	5	5	4	5	> 50 %	2,11
14	61	f	160	65.8	25.7	no	4	3	44	L5	S1	3	5	1	5	> 50 %	2,11
	67.6 ± 11.6^a^	21.4 %	164.5 ± 11.2^a^	81.8 (70.5, 91.4)^b^	32.1 ± 6.7^a^	42.9 %	8.0 (6.0, 9.0) 3^b^	6.5 (4.5, 8.0)^b^	49.3 ± 15.7^a^			5 (3, 5)^b^	5 (5, 5)^b^	3 (1, 4)^b^	5 (5, 6)^b^		

ID: Study patient identification number; BMI: Body mass index; VASback: Visual analogue score for back pain (1–10); VASleg: Visual analogue score for leg pain (1–10); ODI: Oswestry disability Index; Ctrl: Control; MC1: Modic type 1 change; DD: Disc degeneration grade; TES: Total endplate score; Control level: Lumbar vertebra of Control sample; Modic type 1 change (MC1) level: Lumbar vertebra of MC1 sample; NA: Not available. For normally distributed data:

a Mean with standard deviation (SD); For not normally distributed data:

b Median with interquartile range (IQR)

**Table 2.** BMSC from MC1 and Control bone marrow were characterized with 11 different assays. CFU-F: Colony forming unit fibroblast; qPCR: Real-time quantitative polymerase chain reaction; ELISA: Enzyme-linked immunosorbent assay; TGF-β1: Transforming growth factor beta 1; FAK: Focal adhesion kinase; p-FAK: Phosphorylated focal adhesion kinase; *n*: Number of patients used for this assay

Assay	Test parameter	Method	*n*

1	Stem cell marker expression	Flow cytometry	6
2	BMSC identity markers	Flow cytometry	5
3	Differentiation capacity	Histology	8–9
	Adipogenic differentiation LEPR+		3
4	Incidence of BMSC	CFU-F	6
5	Gene expression	Bulk RNA sequencing	4
6	Gene expression	qPCR	5–9
7	Pro-collagen type I alpha 1 synthesis	ELISA	6
8	Collagen contraction	In vitro functional assay	7
9	α-SMA, FAK/p-FAK synthesis	Western Blotting	7–10
10	Adhesion capacity	In vitro functional assay	8–11
11	TGF-β1 stimulated gene expression	qPCR	5
	TGF-β1 stimulated α-SMA synthesis	Western Blotting	5
	TGF-β1 stimulated collagen contraction	In vitro functional assay	5
	TGF-β1 stimulated α-SMA synthesis (LEPR+)	Western Blotting	3–4

**Table 3:** Conjugated antibodies used for flow cytometry.

Antibody	Manufacturer	Dye

CD105	Biolegend	PE
CD90	Abcam	FITC
CD73	Biolegend	APC
CD45	Biolegend	PerCp
CD34	Biolegend	BV421
CD14	Biolegend	APC/Cy7
CD19	Biolegend	BV605
CD54	ThermoFisher	PE
CD106	Biolegend	PE
CD140a	ThermoFisher	PE
CD146	ThermoFisher	PE
CD271	Biolegend	PE
LEPR	Milteny	APC-Vio770
NG2	ThermoFisher	PE
CXCL12	R&D Systems	PE
Nestin	Biolegend	PE

PE: phycoerythrin; FITC: fluorescein; PerCP: peridinin-chlorophyll-protein; BV: brilliant violet; APC: allophycocyanin; Cy7: cyanin 7.

**Table 4.** Primer pairs used for SYBR green qPCR.

Target	Forward primer (5’-3’)	Reverse Primer (5’-3’)

*HPRT1*	AGAATGTCTTGATTGTGGAAGA	ACCTTGACCATCTTTGGATTA
*ACTA2*	GACAATGGCTCTGGGCTCTGTAA	ATGCCATGTTCTATCGGGTACTT
*COL1A1*	CCGATGGATTCCAGTTCGAG	GGTAGGTGATGTTCTGGGAG
*MMP9*	GGATACAGTTTGTTCCTCGT	CTCAGTGAAGCGGTACATAG
*FN1*	TACACTGGGAACACTTACCG	CCAATCTTGTAGGACTGACC
*ITGA1*	GGTTCCTACTTTGGCAGTATT	AACCTTGTCTGATTGAGAGCA
*ITGA2*	GGAACGGGACTTTCGCAT	GGTACTTCGGCTTTCTCATCA
*ITGA3*	AAGGGACCTTCAGGTGCA	TGTAGCCGGTGATTTACCAT
*ITGA4*	GCTTCTCAGATCTGCTCGTG	GTCACTTCCAACGAGGTTTG
*ITGA5*	GTCGGGGGCTTCAACTTAGAC	CCTGGCTGGCTGGTATTAGC
*ITGA6*	GAGCTTTTGTGATGGGCGATT	CTCTCCACCAACTTCATAAGGC
*ITGA7*	CTGTTTCAGCTACATTGCAGTC	GCCTGGTGCTTGGGTTCT
*ITGA8*	AAAAGCAGACGGAAGTGGCT	AGCAGCAACTGAGTATCCAAGG
*ITGA9*	CAAAGGCATCGGCAAGGTTT	TCCCCATTCAGGTCAACTGC
*ITGA10*	TGTTCTTGCCCCTGGTGTTC	CCAGCATCCATCGCTGTCC
*ITGA11*	GGAGGAAGACTTGCGTCG	CACAGGTTCCCCAGTAGATG
*ITGAV*	CTACCTCTGTGCCGCGCCTT	CCCACGAGAAGAAACATCCGGGAAG
*ITGB1*	GCCTTACATTAGCACAACACC	CATCTCCAGCAAAGTGAAAC
*ITGB2*	TTCGGGTCCTTCGTGGACA	ACTGGTTGGAGTTGTTGGTCA
*ITGB3*	TCCAGAGGAAGGGACACCAA	GCAGAGGTACAGATGACCCG
*ITGB5*	GGAGCCAGAGTGTGGAAACA	GAAACTTTGCAAACTCCCTC
*ITGB7*	GCACGCACCTATGTGGAAAC	TCCCAAGCCGTAGTGGTAGA
*ITGB8*	AATTTGGTAGTGGAAGCCTATC	GTCACGTTTCTGCATCCTTC

*HPRT1*: Hypoxanthine phosphoribosyltransferase; *ACTA2*: Alpha smooth muscle actin; *COL1A1*: Alpha-1 type I collagen; *MMP9*: Matrix metallopeptidase 9; *FN1*: Fibronectin; *ITGA*: Integrin subunit alpha; *ITGB*: Integrin subunit beta

**Table 5.** Changes in the expression of bone marrow stromal cell (BMSC) surface markers.

Protein code	Protein name	ΔMFI (MC1-Ctrl)	IQR	*p*

LEPR	Leptin receptor	1395	42, 1724	0.188
CD146	Melanoma cell adhesion molecule (MCAM)	−231	−403, 482	1.000
CD271	Low-affinity Nerve Growth Factor Receptor	−157	−265, 1811	1.000
CXCL12	Stromal cell-derived factor 1 (SDF-1)	75	47, 217	0.063
CD54	Intercellular Adhesion Molecule 1 (ICAM-1)	43	−508, 158	1.000
Nestin	Nestin	1081	974, 3329	0.625
NG2	Neural/glial antigen 2	−457	−1313, 41	0.625
CD140a	Platelet-derived growth factor receptor A (PDGFRA)	−810	−1116, 88	0.625
CD106	Vascular cell adhesion molecule 1 (VCAM-1)	−43	−441, 83	0.625

Difference (MC1 – Ctrl) of median fluorescence intensity (ΔMFI) with inter quartile range (IQR) in flow cytometric analysis are indicated. CD: cluster of differentiation; LEPR: Leptin receptor; NG2: Neural/glial antigen 2

**Table 6:** Dysregulated genes of the GO class “extracellular matrix” (Metacore).

Symbol	Gene name	log_2_ fold-change MC1 vs. Ctrl

*COL22A1*	Alpha-1 type XXII collagen	1.545
*COL4A5*	Alpha-5 type IV collagen	1.15
*WNT2*	Wingless-type MMTV integration site family, member 2	1.037
*HAI2*	Kunitz-type protease inhibitor 2	0.832
*KAL1*	Anosmin-1	0.826
*BMP3B*	Bone morphogenetic protein 3B	0.822
*LRRC15*	Leucine rich repeat containing 15	0.794
*SERPINA3*	Alpha 1-antichymotrypsin	0.792
*ADAMTS16*	A disintegrin and metalloproteinase with thrombospondin motifs 16	0.751
*COL18A1*	Alpha-1 type XVIII collagen	0.746
*PLAT*	Tissue plasminogen activator	0.746
*COL1A1*	Alpha-1 type I collagen	0.676
*FBLN2*	Fibulin-2	0.656
*FBLN5*	Fibulin-5	0.649
*GREM1*	Gremlin-1	0.644
*PCOLCE2*	Pro-collagen C-endopeptidase enhancer 2	0.633
*LAMB3*	Laminin subunit beta-3	0.628
*SERPINE2*	Serpin Peptidase Inhibitor, Clade E, Member 2	0.612
*COL4A2*	Alpha-2 type IV collagen	0.602

*TNC*	Tenascin-C	−0.638
*FGFR2*	Fibroblast growth factor recpetor 2	−0.666
*LUM*	Lumican	−0.69
*CCBE1*	Collagen and calcium-binding EGF domain-containing protein 1	−0.717
*TIG2*	Tazarotene-induced gene 2 protein	−0.747
*COL11A2*	Alpha-2 type XXI collagen	−0.748
*HPSE*	Heparanase 1	−0.841
*CHI3L1*	Chitinase-3-like protein 1	−0.867
*CPXM2*	Carboxypeptidase X, M14 family member 2	−1.188

**Table 7:** Gene expression analysis of integrin subunits.

Gene	mean log_2_fc	sd log_2_fc	p-value

*ITGA1*	0.120	0.605	0.586
*ITGA2*	0.100	0.520	0.609
*ITGA3*	−0.340	1.179	0.447
*ITGA4*	0.000	0.768	0.989
*ITGA5*	0.130	0.616	0.561
*ITGA6*	−0.350	0.897	0.306
*ITGA7*	0.080	1.087	0.832
*ITGA8*	0.908	2.047	0.450
*ITGA10*	0.347	0.769	0.243
*ITGA11*	0.170	0.704	0.514
*ITGAV*	0.225	0.466	0.215
*ITGB1*	0.290	0.306	0.022*
*ITGB2*	−0.010	1.404	0.984
*ITGB3*	−0.041	0.395	0.779
*ITGB5*	−0.018	0.419	0.906
*ITGB8*	−0.150	0.939	0.661

Mean and SD of Log_2_ fold change (Log_2_fc) are indicated.

* *p* < 0.05.

## List of Abbreviations

ACTA2: actin alpha 2, smooth muscle
bFGF: basic fibroblast growth factor
BMI: body mass index
BMSCs: bone marrow stromal cells
BSA: bovine serum albumin
CD: cluster of differentiation
CFU-F: colony-forming unit fibroblast
*COL1A1*: collagen type I alpha 1
*COL4A5*: collagen type IV alpha 5
CXCL12: C-X-C-motif-chemokine 12
DEG: differentially expressed gene
ECM: extracellular matrix
EDTA: ethylenediaminetetraacetic acid
ELISA: enzyme-linked immunosorbent assay
EMT: epithelial-myofibroblast transformation
FACS: fluorescence-activated cell sorting
FAK: focal adhesion kinase
FCS: foetal calf serum
*FN1*: fibronectin
GAG: glycosaminoglycan
GO: gene ontology
GSEA: gene set enrichment analysis
HEPES: 2-[4-(2-hydroxyethyl)piperazin-1-yl] ethanesulfonic acid
HLA: human leukocyte antigen
*HPRT1*: hypoxanthine phosphoribosyltransferase 1
HRP: horseradish peroxidase
ID: study patient identification number
IQR: inter quartile range
LEPR: leptin receptor
LEPR^high^: leptin receptor high
LBP: low-back pain
MC: Modic changes
MC1: Modic type 1 changes
MFI: median fluorescence intensity
micro-CT: micro-computed tomography
*MMP9*: matrix metallopeptidase 9
MRI: magnetic resonance imaging
MSCs: mesenchymal stem cells
NG2: neural/glial antigen 2
NES: normalised enrichment score
ODI: Oswestry disability index
PBS: phosphate-buffered saline
p-FAK: phosphorylated FAK
PMT: pericyte-myofibroblast transition
P/S: penicillin/streptomycin
PVDF: polyvinylidene difluoride
qPCR: quantitative polymerase chain reaction
RT-qPCR: real time qPCR
SD: standard deviation
SDS-PAGE: sodium dodecyl sulphate-polyacrylamide gel electrophoresis
STIR: short tau inversion recovery
TBS: tris-buffered saline
TGF-β1: transforming growth factor beta 1
T1w: T1-weighted
T2w: T2-weighted
VAS: visual analogue score
VASback: visual analogue score for back pain
VASleg: visual analogue score for leg pain
αMEM: minimum essential medium alpha modification
α-SMA: alpha smooth muscle actin
